# Comparative analysis of cervical spine pain and mobility in car *versus* motorbike drivers: a cross-sectional study

**DOI:** 10.7717/peerj.21049

**Published:** 2026-04-09

**Authors:** Aafreen Aafreen, Abdur Raheem Khan, Ashfaque Khan, Ausaf Ahmad, Abdullah Alzahrani, Abdullah Ibrahim Alhusayni, Bsmah H. Alfaifi, Salma O. Naseeb, Ahmed Ghazwani, Ramzi A. Alajam, Mohammed Alshehri, Mohammad Abu Shaphe

**Affiliations:** 1 Department of Physiotherapy, Faculty of Medicine and Health Sciences, Integral University Lucknow, Lucknow, Uttar Pradesh, India; 2 Department of Community Medicine, Kalyan Singh Government Medical College, Bulandshahr, Uttar Pradesh, India, Bulandshahr, Uttar Pradesh, India; 3 Department of Health Rehabilitation Sciences, College of Applied Medical Sciences, Shaqra University, Shaqra, Saudi Arabia; 4 Department of Physical Therapy, College of Nursing and Health Science, Jazan University, Jazan, Saudi Arabia

**Keywords:** Ergonomics, Neck pain, Cervical mobility, Car drivers, Motorbike drivers

## Abstract

**Background:**

Cervical spine pain, particularly among car and motorbike drivers, is a common musculoskeletal issue due to prolonged static postures and repetitive neck movements. This study investigates the prevalence and correlation between cervical spine pain and mobility in car and motorbike drivers.

**Methods:**

In this cross-sectional study, conducted at Integral Hospital and Research Centre, India, 100 participants (50 car drivers and 50 motorbike drivers) were randomly selected with individuals reporting cervical pain. Participants completed demographic information, driving duration, and cervical pain severity and underwent cervical mobility assessments using smartphone-based tools. Statistical analysis was conducted using independent t-tests and Pearson’s correlation with 95% confidence intervals and Cohen’s d effect sizes, at a significance level of *p* ≤ 0.05.

**Results:**

Car drivers (mean age 39.2, driving experience 15.6 years, 70% male) and motorbike drivers (mean age 34.8, experience 12.4 years, 80% male) were studied. Motorbike drivers reported higher cervical pain on visual analogue scale (VAS) 5 compared to car drivers VAS 4. Inverse correlations between cervical pain and mobility were noted, with motorbike drivers showing significantly lower cervical mobility across all movements. Negative correlations varied in strength across different movements for both groups, generally more substantial for car drivers.

**Conclusion:**

This study highlights the association between cervical pain and reduced mobility among drivers, particularly motorbike drivers. Although causality cannot be determined, the findings support ergonomic interventions and driver education to promote better postures and musculoskeletal health.

## Introduction

Cervical spine pain is a prevalent musculoskeletal problem affecting a large proportion of the global population, with a lifetime prevalence estimated to be between 30% and 50% (Hoy et al., 2014). Patients with cervical spine pain may present with a wide range of symptoms including localized pain, radiculopathy, dizziness, vertigo, headaches, palpitations, tinnitus, blurred vision, and even gastrointestinal discomfort (Chu, Bhaumik & Lo, 2020; Olson & Joder, 2001; Sial et al., 2024). Its impact extends beyond physical discomfort, often contributing to depression, absenteeism, reduced work productivity, and increased healthcare utilization (Fejer, Kyvik & Hartvigsen, 2006). Car and motorbike drivers, are particularly susceptible due to prolonged static postures, vibration exposure, and repetitive neck movements (Porter & Gyi, 2002).While both groups experience extended periods of driving, their postural requirements vary, car drivers generally maintain a supported, semi-reclined posture, whereas motorbike drivers often assume a more dynamic, upright position with increased demands on neck movement. This biomechanical difference may have noticeable effects on neck health and mobility (Jakobsson, Isaksson-Hellman & Lindman, 2008; Das & Maurya, 2019).

Previous studies have highlighted the variable prevalence of neck pain among drivers, influenced by cultural, occupational, and ergonomic factors (Guzman et al., 2008; Aafreen et al., 2023a). However, comparative data on cervical spine pain and mobility between car and motorbike drivers remain limited.

Despite the potential implications of neck pain on drivers’ health and well-being, the current literature lacks comparative studies evaluating cervical spine pain and mobility specifically between car and motorbike drivers. Previous studies have highlighted notable differences in neck pain prevalence between these groups, primarily because of variations in driving posture and exposure conditions. Motorbike riders have been reported to experience a higher frequency of neck and upper back pain up to 58% than car drivers (approximately 40%) (Aafreen et al., 2023b). This discrepancy may be attributed to several factors unique to motorcycle riding, including greater exposure to whole-body vibration, the forward-leaning posture required for balance and control, and environmental stressors such as wind resistance and temperature fluctuations.

The cervical range of motion is essential for normal function and is frequently reduced in individuals with neck pain (Aafreen et al., 2023b). While prior research has examined risk factors such as driving duration, ergonomics, and psychological stress, the relationship between pain severity and mobility, especially in motor vehicle drivers, remains underexplored (Kazeminasab et al., 2022).This study addresses this gap by examining cervical spine pain and mobility in car and motorbike drivers, aiming to provide insights into targeted interventions that may improve driver health and safety.

Therefore, this study hypothesized that motorbike drivers experienced a higher prevalence and greater severity of cervical spine pain, along with more restricted cervical mobility, compared to car drivers. Furthermore, it was predicted that increased neck pain severity was negatively associated with cervical range of motion in both groups.

## Materials & Methods

### Study design and population

This cross-sectional study, conducted in accordance with the STROBE guidelines, was carried out from November 2022 to March 2023 in the physiotherapy division of Integral Hospital and Research Centre, part of Integral University in Lucknow, Uttar Pradesh, India. The Institutional Ethical Committee of Integral University, Lucknow, India, provided ethical authorization for this study (reference number: IEC/IIMSR/2022/70). The study was registered under the Clinical Trial Registry India (CTRI) with registration number CTRI/2022/11/047689. The study was conducted in accordance with the 1964 Helsinki Declaration, and all participants provided written consent before participating (World Medical Association, 2013).

The study included the assessment of neck pain in a consecutive sample of one hundred patients visiting the Outpatient Department (OPD). The sample size determination was conducted as previously used formula *n* = *z*^2^*p*(1 − *p*)/*d*^2^, described in Akhtar, Mumtaz & Khan (2025), Daniel (1999). Where *n* = sample size, *p* = 50% (assuming maximum variability), power (1−β) = 0.80, alpha level (α) = 0.05 (corresponding to a 95% confidence interval; *Z* score = 1.96), margin of error (d) = 10%, and non-response rate = 10%. This approach aimed to achieve a minimum acceptable intraclass correlation coefficient (ICC) of 0.60. Based on these parameters, a minimum of 100 participants was determined to be necessary to detect a statistically significant difference within the study parameters. Therefore, stratified random sampling was employed to select participants, thereby ensuring representation across diverse categories, including age, gender, and type of vehicle driven. We enrolled 100 participants who reported neck pain (50 car drivers and 50 motorbike drivers).

The inclusion criteria for participation were a valid driving license, at least three years of driving experience. Participants were selected using simple random sampling, targeting drivers from various socio-economic backgrounds and geographical locations. Both male and female riders were represented in each group. Participants had to be aged between 25 and 58 and drive a car or motorbike for at least two hours daily to qualify for either group (Von Elm et al., 2007). Neck pain refers to discomfort in the cervical spine region, arising from conditions like muscle strain, ligament injury, nerve compression, or degenerative changes (Childs et al., 2008).

Participants regardless of whether they had or had not received professional treatment for neck pain, if their perceived neck pain intensity, as measured on a 0–10 verbal numerical pain rating scale (0: no pain; 10: worse pain possible), was between 2 to 7 were included. Subjects were excluded if they had a history of cervical spine surgical intervention, any neurological disorders, or any other medical conditions affecting cervical spine mobility.

### Procedure

Participants were asked to complete a self-administered questionnaire assessing demographic information (*e.g.*, age, gender, occupation), driving habits (*e.g.*, average daily driving time, driving posture, use of ergonomic devices), and self-reported neck pain severity on a Visual Analogue Scale (VAS) of 0 (no pain) to 10 (worst pain imaginable) (Schaufele & Boden, 2003).

Cervical mobility was assessed using the smartphone clinometer and compass, which is a non-invasive, reliable, and valid tool for measuring cervical spine movement. The Clinometer (Peter Breitling, Version 3.3, http://www.plaincode.com/products) is an application developed for smartphones that makes use of the device’s three inbuilt accelerometers (LIS302DL accelerometer) to quantify cervical spine ranges of motion in the frontal and sagittal planes. The smartphone’s built in Compass app is used to assess cervical ranges of motion in the horizontal plane. The program uses the smartphone’s magnetometer, which detects orientation concerning the Earth’s magnetic field using the Hall Effect (http://www.memsjournal.com/2011/02/motion-sensing-in-the iPhone-10 electroniccompass.html).

Subjects were instructed to perform a series of cervical spine ranges of motion, including flexion, extension, lateral flexion (right and left), and rotation (right and left), to their maximum comfortable range. The exact sequence of cervical range-of-motion tests was administered to all subjects. Subjects were asked to actively perform all six cervical ranges of motion in the prescribed order before testing to minimize creep and get used to the process. All participants received uniform verbal instructions, and each underwent a single-session assessment to minimize variability. A single trained assessor conducted all evaluations using the same instruments and protocols to ensure the intra-rater reliability. Cervical mobility and VAS measurements were recorded using an iPhone 15 with standardized app settings, providing objective data in degrees for each movement (Khan et al., 2023; Aafreen et al., 2023c).

### Statistical analysis

The statistical analysis was conducted using SPSS Statistics software version 26.0 (IBM Corp., Armonk, NY, USA). Descriptive statistics were used to summarize the data. Independent t-tests were employed to compare the means of two independent groups and determine whether the groups had a significant difference. For each cervical movement including flexion, extension, lateral flexion (right and left), and rotation (right and left) mean differences, 95% confidence intervals (CI), *t*-values, *p*-values, and Cohen’s d effect sizes were reported to assess both statistical and practical significance. Cohen’s d values were interpreted as small (0.2), medium (0.5), and large (0.8). A *p*-value ≤ 0.05 was considered statistically significant. Pearson’s correlation coefficients, along with 95% confidence intervals (calculated using Fisher’s z-transformation), and scatter plots were used to examine the association between cervical spine pain severity and mobility. No corrections for multiple comparisons were applied due to the exploratory nature of the study. Missing data were handled using listwise deletion, and only complete cases were included in the analysis.

## Results

The demographic characteristics and driving habits of the subjects are represented in Table 1. The mean age of car drivers was 39.2 years, slightly higher than that of motorbike drivers (34.8 years). The majority of participants were male, with 70% of car drivers and 80% of motorbike drivers being male. Car drivers had more driving experience on average (15.6 years) than motorbike drivers (12.4 years). Weekly driving hours were similar between the two groups, with car drivers spending 15.1 h per week and motorbike drivers spending 14.2 h per week on average. The mean neck pain severity was slightly higher among motorbike drivers (5.0) compared to car drivers (4.3), with a prevalence of 76% and 68%, respectively.

**Table 1:** Demographic characteristics, driving habits, and self-reported neck pain severity of participants (*N* = 100).

**Variable**	**Car drivers (*N* = 50)**	**Motorbike drivers (*N* = 50)**	**Total** **(*N* = 100)**	***p*-value**
Age (years)	39.2 ± 10.4	34.8 ± 9.7	37.0 ± 10.2	0.288^*^
**Gender, n (%)**				
Male	35 (70)	40 (80)	75 (75)	0.248^#^
Female	15 (30)	10 (20)	25 (25)	
Driving experience (years)	15.6 ± 8.2	12.4 ± 7.9	14.0 ± 8.1	0.049^*^
Weekly driving hours	15.1 ± 6.4	14.2 ± 5.9	14.7 ± 6.2	0.530^*^
Neck pain severity VAS (0–10)	4.3 ± 2.5	5.0 ± 2.7	4.7 ± 2.6	0.181^*^
Prevalence of neck pain, n (%)	34 (68)	38 (76)	72 (72)	0.372^#^

**Notes.**

VAS, Visual Analogue Scale, Values are expressed as mean ± standard deviation.

* Chi-square test.

# Independent *t*-test.

Table 2 displays correlation between neck pain severity and cervical mobility for both car drivers and motorbike drivers. Negative *r* values indicate that higher pain scores correspond to reduced mobility, with correlations ranging from weak to moderate (*r* = −0.190 to −0.672).

**Table 2:** Pearson’s correlation coefficients (r) between neck pain severity and cervical mobility.

**Cervical movement**	**Car drivers (*n* = 50)**	**Motorbike drivers (*n* = 50)**
	*r* (95% CI), *p*-value	*r* (95% CI), *p*-value
Flexion	−0.341 [−0.566, −0.069], *p* = 0.015	−0.408 [−0.616, −0.146], *p* = 0.003
Extension	−0.402 [−0.612, −0.139], *p* = 0.004	−0.215 [−0.465, 0.067], *p* = 0.133
Right lateral flexion	−0.582 [−0.740, −0.362], *p* < 0.001	−0.672 [−0.801, −0.484], *p* < 0.001
Left lateral flexion	−0.575 [−0.736, −0.353], *p* < 0.001	−0.190 [−0.445, 0.093], *p* = 0.186
Right rotation	−0.634 [−0.775, −0.432], *p* < 0.001	−0.337 [−0.563, −0.065], *p* = 0.017
Left rotation	−0.390 [−0.603, −0.125], *p* = 0.005	−0.315 [−0.546, −0.040], *p* = 0.026

**Notes.**

*r*, Pearson’s correlation coefficient; CI, Confidence Interval; *p*, *p*-value.

For flexion, car drivers display a weak negative correlation (*r* = −0.341, *p* = 0.015), while Motorbike Drivers exhibit a stronger, moderate negative correlation (*r* = −0.408, 95% CI [−0.616 to −0.146], *p* = 0.003). Extension shows a moderate negative correlation for car drivers (*r* = −0.402, *p* = 0.004) and a weaker, non-significant correlation for motorbike drivers (*r* = −0.215, *p* = 0.133). For right lateral flexion, both car drivers and motorbike drivers exhibit a strong negative correlation (Right lateral flexion *r* = −0.582, 95% CI [−0.740 to −0.362], *p* < 0.001) and *r* = −0.672, 95% CI [−0.801 to −0.484], *p* < 0.001), respectively). In terms of left lateral flexion, car drivers show a strong negative correlation (*r* = −0.575, 95% CI [−0.736 to −0.353], *p* < 0.001), indicating a significant relationship between left lateral flexion and decreased neck pain severity. However, for motorbike drivers, the correlation is weaker and not statistically significant (*r* = −0.190, *p* = 0.186). Right rotation demonstrates a strong negative correlation for both car drivers (*r* = −0.634, 95% CI [−0.775 to −0.432], *p* < 0.001) and motorbike drivers (*r* = −0.337, *p* = 0.017), implying that an increase in right rotation is associated with a decrease in neck pain severity. Left rotation also shows moderate negative correlations for both car drivers (*r* = −0.390, *p* = 0.005) and Motorbike Drivers (*r* = −0.315, *p* = 0.026).

Table 3 displays no statistically significant differences in cervical mobility or pain severity (VAS) between car and motorbike drivers (all *p* > 0.05, *df* = 98), greater pain and restricted mobility in motorbike drivers due to dynamic postures. The VAS mean difference (−0.700; 95% CI [−1.504–0.104]; *p* = 0.087) suggests motorbike drivers experience moderately higher pain (Cohen’s d = −0.345, small effect), consistent with their reported mean VAS of 5 *versus* 4 for car drivers.

**Table 3:** Independent *t*-test comparisons of cervical mobility scores between car and motorbike drivers.

**Variable**	**Mean difference (95% CI)**	***t* (*df* = 98)**	***p*-value**	**Cohen’s d (effect size)**
VAS score	−.700 (−1.504–0.104)	−1.727	0.087	−0.345 (small)
Flexion	3.280 (−.144–6.704)	1.901	0.060	0.380 (small-medium)
Extension	.940 (−2.035–3.915)	0.627	0.532	0.125 (small)
Right lateral flexion	.000 (−1.130–1.130)	0.000	1.000	0.000 (none)
Left lateral flexion	.900 (−.692–2.492)	1.122	0.265	0.224 (small)
Right rotation	.860 (−2.146–3.866)	0.568	0.572	0.114 (small)
Left rotation	1.160 (−1.158–3.478)	0.993	0.323	0.199 (small)

**Notes.**

VAS, Visual Analogue Scale; CI, Confidence Interval; df, degrees of freedom; *p*, *p*-value; Cohen’s d indicates the effect size.

Mobility comparisons reveal a near-significant advantage for car drivers in flexion (mean difference: +3.280°; 95% CI [−0.144–6.704]; *p* = 0.060; *d* = 0.380, small-medium), implying ∼3° forward flexion. Other movements extension (+0.940°), lateral flexions (∼0-−0.900°), and rotations (∼0.860–1.160°)—show negligible differences (*p* = 0.265–1.000; *d* < 0.225, none to small), with symmetric right/left patterns indicating no asymmetric biases from driving habits.

**Figure 1:**
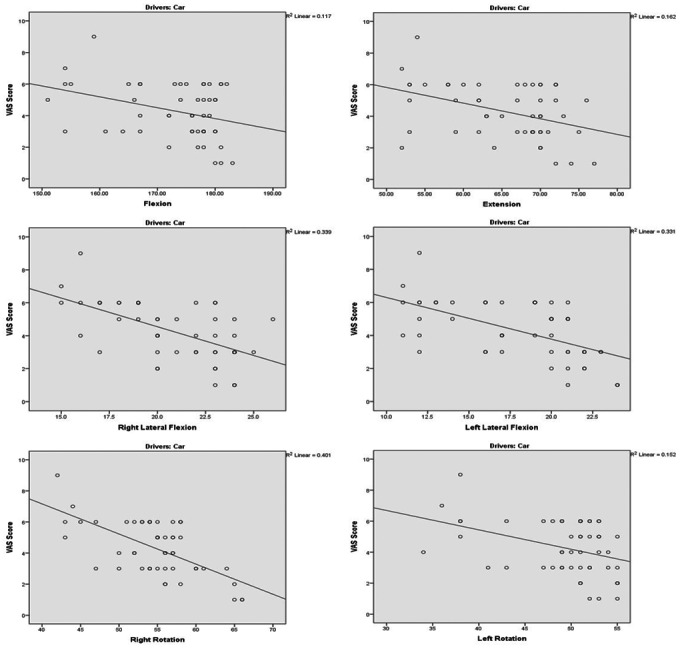
Scatter plot between neck pain severity and cervical mobility in car drivers. Scatter plots illustrating the relationship between Visual Analogue Scale (VAS) scores and cervical range of motion (ROM) in car drivers (*n* = 50). Each plot shows a linear regression line and *R*^2^ value for the correlation between VAS scores and the cervical movements.

**Figure 2:**
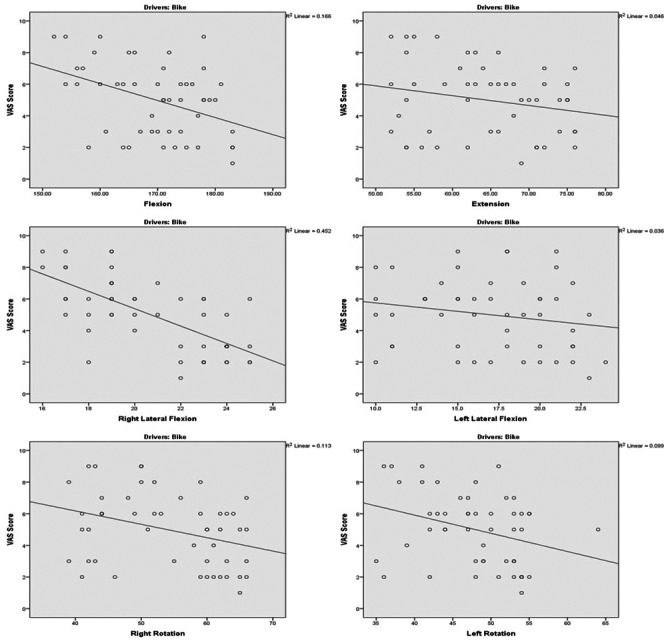
Scatter plot between neck pain severity and cervical mobility in bike drivers. Scatter plots depicting the relationship between Visual Analogue Scale (VAS) scores and cervical range of motion (ROM) in motorbike drivers (*n* = 50). Each plot includes a linear regression line and the coefficient of determination (*R*^2^) for the cervical movements.

In a scatter plot (Figs. 1 & 2), the R-square value, also known as the coefficient of de-termination, R-square value showed that variability in the pain level can be explained by the cervical mobility.

## Discussion

The present study findings illuminate on comparative analysis of cervical spine pain and mobility problems among car and motorbike drivers. Our research shows that motorbike drivers more commonly report neck pain and experience slightly more severe symptoms than car drivers. However, it is important to note that most of the between-group comparisons in cervical mobility were not statistically significant, though they may be clinically relevant. Therefore, these findings should be interpreted with caution, emphasizing the observed patterns rather than confirmed group differences.

In contrast to the non-significant between group mean differences, correlation analyses revealed meaningful relationships. We found a strong negative correlation between neck pain severity and cervical mobility, suggesting that a higher pain intensity is associated with reduced cervical motion. This correlation, observed across several movement planes, underscores the potential clinical relevance of pain and cervical mobility interactions in both driver groups. These findings are particularly important given the increasing global reliance on both cars and motorbikes for transportation and the need to better understand their impact on musculoskeletal health.

From a clinical and public health perspective, these results highlight the importance of preventive ergonomic interventions, particularly for motorbike drivers who may be more susceptible to cervical spine disorders due to altered driving biomechanics. This insight is essential for physiotherapists and occupational health professionals aiming to develop targeted therapeutic intervention or ergonomic strategies for this population. For motorbike drivers, this may include adjusting the handlebar height, using cushioned and vibration-absorbing seats, and wearing posture-supportive gear. Car drivers may benefit from lumbar-cervical support cushions and adjustable headrests to encourage proper spinal alignment. Regular driving breaks, postural education, and neck mobility exercises should also be integrated into occupational health programs.

Our correlation findings align with prior literature, which also reported a negative association between neck pain severity and cervical mobility (Oleksy et al., 2021; O’Leary et al., 2009). Reduced mobility has been linked to muscle imbalances and impaired joint function that increase stress on the cervical spine (Multanen et al., 2021; Liao, Chen & Ge, 2022). The specific challenges faced by motorbike drivers, such as prolonged static postures and repetitive neck movements, are consistent with past findings linking these factors to increased neck pain severity (Vishal et al., 2023).

Demographic patterns observed in our study also reflect previous research. Males were more frequently involved in both car and motorbike driving, consistent with earlier studies (Jiménez-Mejías et al., 2014; Cordellieri et al., 2016). Differences in driving experience may also influence cervical spine pain, as greater experience might relate to better posture and habits (Muslim et al., 2021).

Although most between-group differences in cervical mobility were not statistically significant, trends suggest that motorbike drivers may be at higher risk due to dynamic postures. However, this study did not assess biomechanical factors in depth. Future research should investigate underlying mechanisms such as altered motor control patterns, proprioceptive deficits, and sensorimotor disturbances, which have been linked to chronic cervical spine pain (Hogan et al., 2020; Takacs et al., 2021; Zheng et al., 2022; Mansfield et al., 2023). Moreover, impaired proprioception has been shown to be associated with chronic cervical spine pain, which could potentially affect cervical mobility (Peng et al., 2021).

Sagittal spinal posture may also play a role. For example, Kim, Davis & Menger (2023) found that spinal posture differed significantly between younger and older adults, influencing cervical mobility and pain. Future studies should consider these biomechanical aspects.

Despite the valuable insights generated by this study, it is important to acknowledge that the use of self-reported pain intensity may introduce response bias, as individual pain perception is subjective and could vary based on potential confounding factors such as psychological, occupational history, physical activity level, or other health conditions. Moreover, the generalizability of the findings is limited by the fact that the study was conducted in a single geographic location, which may not represent broader populations with different driving environments, ergonomic conditions or cultural habits. This study used a cross-sectional design, which prevents conclusions about the causality between neck pain and cervical mobility. Additionally, the sample may not fully represent the diverse demographics of all drivers and recall bias may have affected responses related to pain and driving habits. The sample size was also relatively small (*n* = 100) and limited to a single geographic region, which may affect the generalizability of the findings to broader populations.

Furthermore, the present study did not examine the underlying mechanisms that might contribute to the observed differences in neck pain severity and cervical mobility between motorbike and car drivers.

Future research should focus on longitudinal designs to assess changes over time and better understand the causal relationships between driving habits, postures, and cervical spine health. Moreover, studies should explore the influence of other symptoms, such as dizziness, headaches, and visual disturbances, which are often associated with chronic neck pain but were not included in the present study. It is also recommended that future studies with larger and more diverse samples across multiple regions to validate and extend these findings.

Furthermore, future research should implement multivariate analysis techniques, such as regression, to control confounding variables and confirm independent relationships.

## Conclusions

Our research provides essential insights into cervical spine pain and mobility among drivers, focusing on motorbike riders. As this study was a cross-sectional study, causal relationships could not be established, and the findings should be interpreted as associations only. Practical implications include promoting ergonomic interventions, such as improved seating posture and regular movement breaks, and educational initiatives aimed at increasing driver awareness of musculoskeletal health.

## Supplemental Information

10.7717/peerj.21049/supp-1 Supplemental Information 1 STROBE checklist

10.7717/peerj.21049/supp-2 Supplemental Information 2 Data

## Additional Information and Declarations

### Data Availability

The following information was supplied regarding data availability: The data is available in the Supplemental File.
